# Circular RNA-Encoded Proteins in Disease Pathogenesis

**DOI:** 10.7150/ijbs.110146

**Published:** 2025-09-29

**Authors:** Jude Uzoechina, Zhijun Zhang

**Affiliations:** 1 Shenzhen Key Laboratory of Precision Diagnosis and Treatment of Depression, The Brain Cognition and Brain Disease Institute of Shenzhen Institute of Advanced Technology, Chinese Academy of Sciences, Shenzhen, Guangdong 518055, P.R. China.; 2 Department of Mental Health and Public Health, Faculty of Life and Health Sciences, Shenzhen University of Advanced Technology, Shenzhen, Guangdong 518055, P.R. China.; 3 University of Chinese Academy of Sciences, Beijing 100049, P. R. China.; 4 Department of Neurology in Affiliated Zhongda Hospital and Jiangsu Provincial Medical Key Discipline, School of Medicine, Institute of Neuropsychiatry, Key Laboratory of Developmental Genes and Human Disease of Ministry of Education, Southeast University, Nanjing, Jiangsu 210009, P. R. China.

**Keywords:** Circular RNAs, Circular RNA-encoded proteins or translatable circular RNAs, pathogenesis, molecular mechanisms, biomarkers, clinical relevance

## Abstract

Circular RNAs (circRNAs) are structurally stable and covalently-linked ring RNA molecules. Due to backsplicing, they are directly joined by phosphodiester linkage, which confers much greater stability relative to their linear mRNA counterparts. Recent studies indicate that circRNAs also encode proteins involved in mechanisms associated with the pathogenesis of various diseases, offering new treatment insights. This review briefly summarizes the history, characteristics, and functions of circRNAs; the formation and translation mechanisms of circRNA-encoded proteins; and computational and experimental techniques for identifying/predicting the protein-encoding potential of circRNAs. We also summarized their role in disease pathogenesis, which includes how they could be targeted and harnessed as novel therapeutic options for disease treatment. Finally, we stated some current limitations to studies on circRNA-encoded proteins and concluded with a discussion of future research directions to facilitate effective clinical translation.

## Introduction

CircRNAs are an intricate class of endogenous, single-stranded (ss) RNA, formed predominantly by the backsplicing of precursor mRNAs (pre-mRNAs) 1, 2. The formation of circRNAs is aided by the complementarity of the RNA sequences at both ends of the RNA transcript 3, 4. Through the process of backsplicing, the downstream 5' splice site (splice donor) of the RNA molecule is joined via a phosphodiester bond to the upstream 3' splice site (splice acceptor), resulting in a covalently closed circRNA molecule 5.

The discovery of circRNAs dates to the mid-1970s when they were described as defective interfering (DI) particles with an uncharacteristic ability to form circular structures, indicating that DI-RNAs possess complementary ends 6. That same year, circRNAs were also reported as viroids, which are single-stranded, highly thermostable infectious RNA molecules that affect higher plants 4. The circular form of ssRNA was initially observed in the cytoplasm of eukaryotic cells, although its functions were largely unknown 3. Within the next decade, yeasts such as *Saccharomyces cerevisiae* were found to contain mitochondrial circRNAs 7. The circRNA genome was first observed in an animal virus: hepatitis delta (δ) 8. In the 1990s, exonic circRNAs were reported as stable, mis-spliced products localized in the cytoplasmic partition in human eukaryotic cells, processed from nuclear precursor mRNAs 9. Subsequently, studies began to emerge on the functional roles of some circRNAs and their abundance in mostly eukaryotic cells 10-12. However, it is very crucial to note that extensive evidence has been provided that the majority of circular RNAs are indeed non-functional and are mainly byproducts of splicing errors 13. Therefore, while some circular RNAs have been proven to possess functional roles, a great majority of them are likely non-functional splicing byproducts and may be detrimental 13.

The circular structure of circRNAs confers an unusual stability compared to their linear counterparts 14, 15. As a result, circRNAs can resist degradation upon reaction with ribonucleases, particularly exonucleases 16. Further studies have shown that circRNAs are highly tissue-specific and significantly enriched in the brain 11. Despite being produced in the nucleus, circRNAs are mostly localized in the cytoplasm. A recent study showed that nuclear export of circRNAs from the nucleus to the cytoplasm is aided by nuclear Ran-GTP, which enhances the interaction of IGF2BP1 with circRNA, recruiting exportin-2 for the export of circRNAs from the nucleus to the cytoplasm 17. During neurogenesis, additional factors necessary for efficient circRNA translocation have been identified. CircRNAs with adenosine (A)-rich motifs were retained in the nucleus through interaction with PABPC and remained trapped by TPR protein - this action was reversed following neuronal differentiation 18.

The development of high-throughput RNA-sequencing technology was a crucial breakthrough for the identification and quantification of novel circRNAs 19. However, due to their complexity, circRNAs remain difficult to characterize. Modern methods rely on RNA-sequencing methodologies for circRNA characterization, which can contribute to false reads and result in the loss of the majority of circRNAs because of their low abundance 20.

At present, some widely known functions of non-coding circular RNAs include the following: acting as sponges for miRNAs 2, thereby regulating gene expression; interacting with RNA binding proteins (RBPs) 21; and acting as transcriptional modulators by binding to and regulating RNA polymerase II (Pol II) 12, 22. Most circRNAs do not contain ORFs or harbor specific IRES sequences, thus, would rarely undergo translation, and have been classified as untranslatable non-coding RNAs. For instance, studies employing ribosome profiling, standard proteomics and extensive analysis across species have shown that even among AUG circRNAs, which are the most conserved and abundant circRNA group, no indication of translation was recorded, thus suggesting that circRNAs are majorly non-coding 23. However, recent works have revealed that some circRNAs actually do encode proteins implicated in notable disease-related pathways, such as the Wnt/β-catenin pathway, hedgehog signaling pathway, and MAPK/ERK pathway 24. Thus, circRNA-encoded proteins have garnered much attention as potential therapeutic targets with available treatment options such as gene therapy approaches, small molecule inhibitors and antibody-based therapies. This review aims to explicitly shed more light on the clinical relevance of circRNA-encoded proteins, reported to play a significant role in the pathogenesis of various diseases ranging from cancer to neurological and cardiac diseases, and suggests that targeting these proteins would provide a fresh outlook and novel perspective towards providing long-lasting treatment for these diseases.

Therefore, this review focuses on known, translatable circRNAs and highlights their role and involvement in the pathogenesis of diseases in relation to protein-protein interactions, while discussing the ongoing challenges associated with profiling circRNA-encoded proteins.

## Formation mechanisms of circRNAs

**Figure 1:**
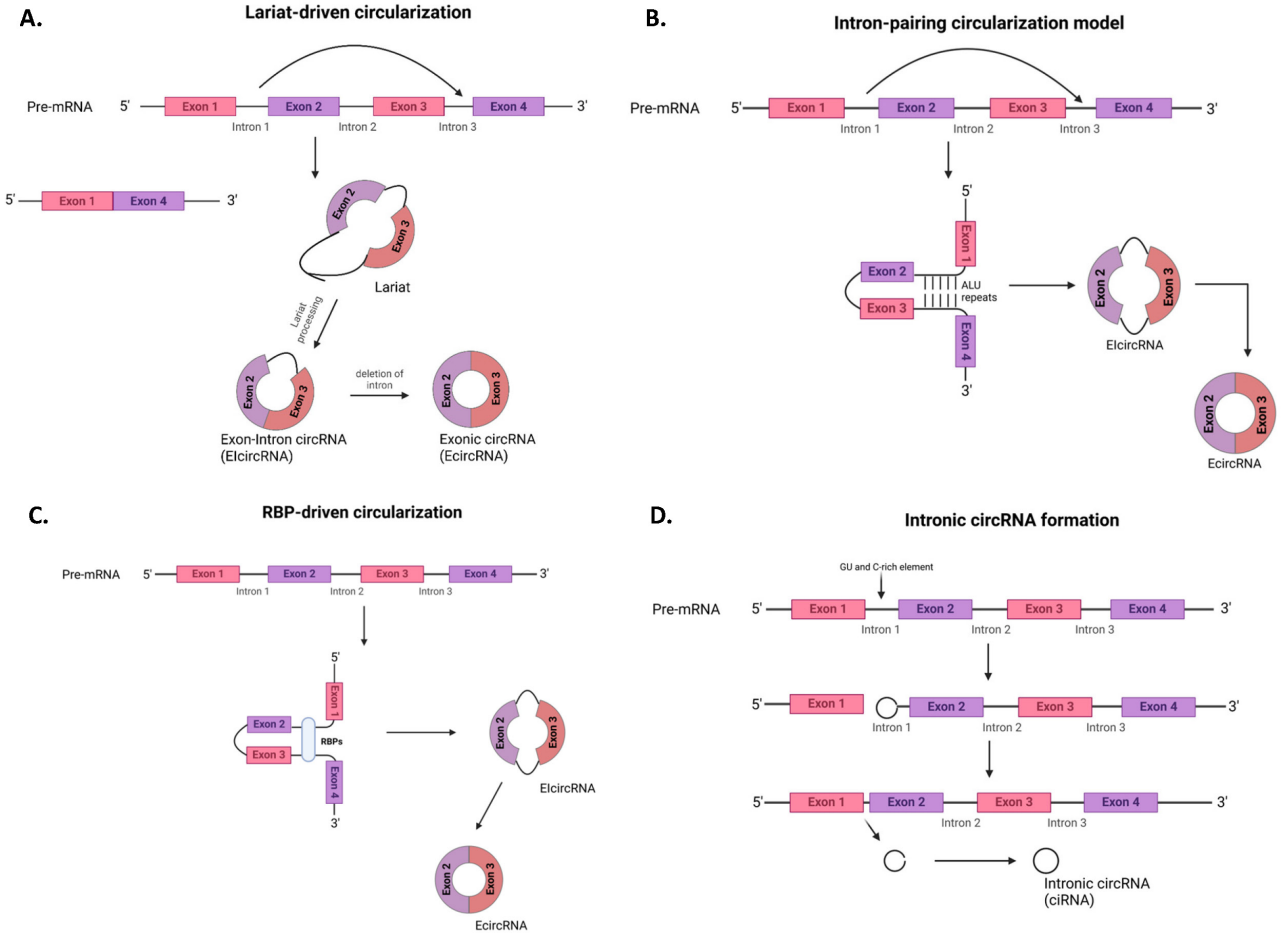
** Formation mechanisms of circRNA.** The different mechanisms for the formation of circular RNAs is shown above; **a)** Lariat-driven circularization model **b**) Intron-pairing circularization model **c)** RBP-driven circularization model **d)** Intronic circRNA formation. RBP, RNA-binding proteins. (Created in https://BioRender.com)

There are three major types of circular RNAs formed by different molecular mechanisms: exonic circular RNAs (EcircRNAs), exon-intron circular RNAs (EIcircRNAs), and intronic circular RNAs (ciRNAs) (Fig. 1) 12, 25, 26.

### Lariat-driven model of circularization (exon-skipping)

EIcircRNAs are formed through the lariat-driven model of circularization commonly known as the exon-skipping method (Fig. 1a) 27. Here, a linear mRNA with one or more skipped exons formed through traditional/canonical splicing gives rise to another RNA molecule called a lariat that contains the skipped exons. Subsequent lariat processing, followed by backsplicing, results in circRNA formation.

### Intron-pairing circularization model

EcircRNAs and EIcircRNAs can be formed by the intron-pairing circularization model through complementarity, which is driven by the *Alu* repeats between adjacent introns located at the 5' and 3' splice sites of pre-mRNAs (Fig. 1b) 27.

### RBP-driven circularization model

The formation of both EIcircRNAs and EcircRNAs can also be driven by the RBP-driven circularization model, which involves the linkage of the splice donor and splice acceptor of pre-mRNA by RBPs such as HNRNPL, MBNL1, FUS, etc. (Fig. 1c) 5, 28.

### Intronic circular RNA (ciRNAs) formation

Finally, ciRNAs formation is aided by the localization of specific sequences, such as a 7 nt GU-rich element near the 5' splice site and an 11 nt C-rich element close to the branch site of the intron lariat (Fig. 1d) 5.

### Comparison of translation mechanisms between circRNAs and mRNAs

CircRNAs were initially thought to be untranslatable or non-coding RNAs due to the absence of the 3' poly A tail and 5' N7 methylguanosine (m7G) cap required for canonical translation of mRNAs into their respective polypeptides. However, recent observations have debunked that idea. Some endogenous circRNAs possess an open reading frame (ORF) together with start and stop codons, which accentuates a broader potential for translation than previously recognized 29. Thus, some circRNAs are indeed translatable and can undergo translation via mechanisms different from the canonical cap-dependent translational mechanism. Importantly, circRNAs have been identified in heavy polysomic fractions, as validated by ribosome fragment analysis, transcriptome-wide ribosome profiling, and polysome profiling data 30.

**Figure 2:**
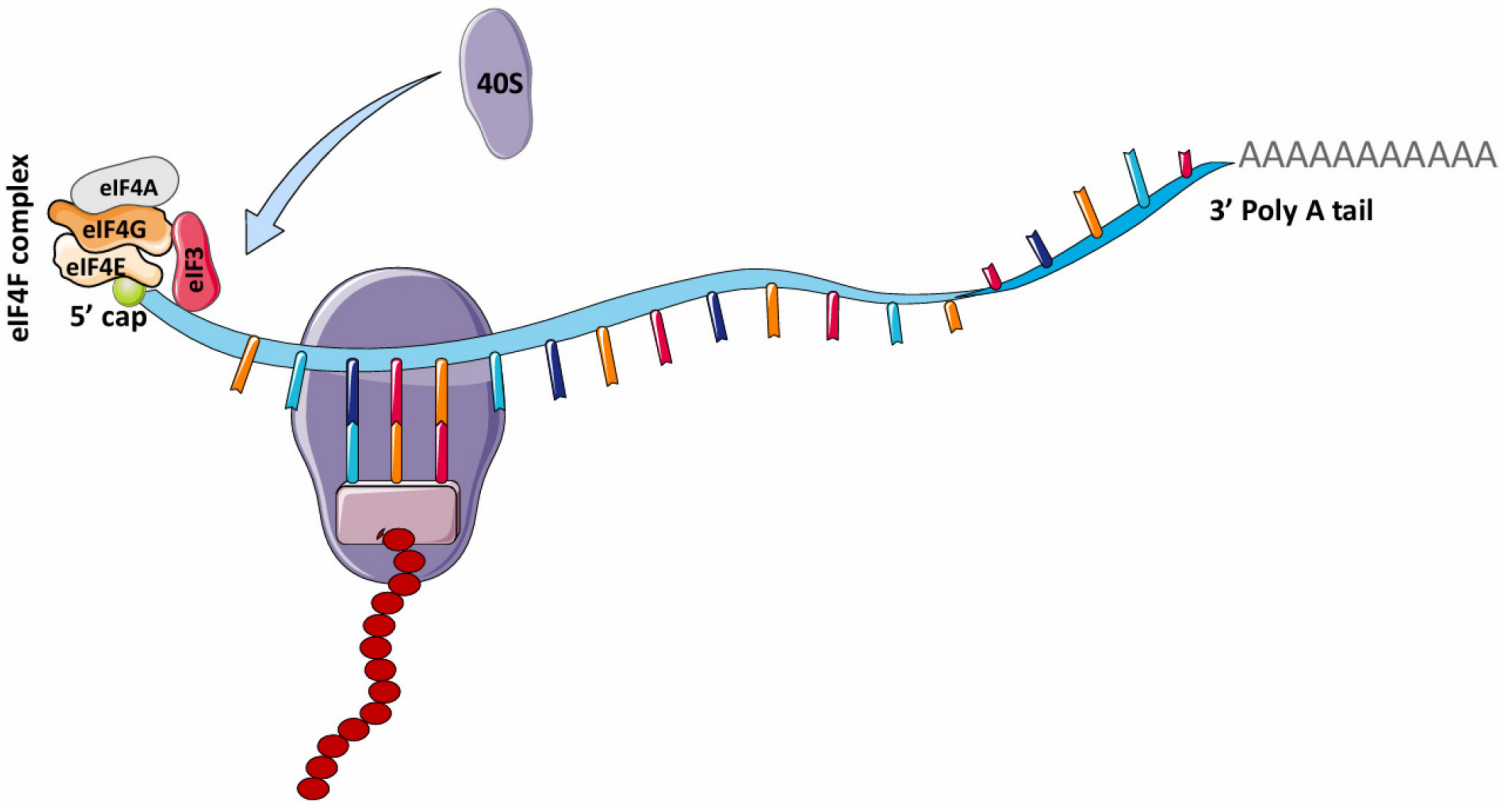
** mRNA translation in eukaryotes.** The attachment of the eIF4F complex (eIF4G, eIF4A, eIF4E) and eIF3 complex to the 5' cap of mRNA is necessary for recruiting 40S ribosome for translation. This is known as the canonical cap-dependent translation. eIF4F, Eukaryotic initiation factor 4F; eIF4G, Eukaryotic initiation factor 4G; eIF4A, Eukaryotic initiation factor 4A; eIF3, Eukaryotic initiation factor 3. (Created in Servier Medical Art)

In 1975, it was reported that the addition of the 5' N^7^-methylguanosine (5' m^7^G) cap to eukaryotic mRNAs by the enzyme, guanine-7 methyltransferase is required to initiate protein synthesis (translation) in various eukaryotic cells, such as reovirus and vesicular stomatitis virus (VSV) mRNAs and reticulocyte mRNAs 31, 32. This finding gave rise to the theory that the canonical cap-dependent translational mechanism is required for eukaryotic mRNA translation. For eukaryotic mRNAs, the traditional process of protein translation involves three major steps: initiation, elongation and termination. In mRNA translation initiation, the 5' m^7^G is recognized by eIF4F (eukaryotic initiation factor 4F), a protein complex comprising eIF4G (which binds eIF4E, eIF4A, and eIF3), eIF4A (an ATP-dependent RNA helicase), and eIF4E (Fig. 2) 33. The binding of eIF4E to the 5' m^7^G structure of mRNA recruits these other factors to form a complex unit that enhances initiation of eukaryotic mRNA translation. During elongation, the ribosome simply travels along the mRNA and catalyzes the transfer and formation of peptide bonds between amino acids in the growing polypeptide chain. Upon termination, the polypeptide unit is released after recognition of the termination codon by the eukaryotic release factor (eRF).

However, this process is quite different for circRNAs and remains poorly understood. Research over the past decade has shown that circRNA can undergo translation through the independent ribosomal entry site (IRES) and non-IRES translation mechanisms. As neither process requires the presence of 5' or 3' ends for translation initiation, these are known as cap-independent translation mechanisms.

### IRES translation mechanism

**Figure 3:**
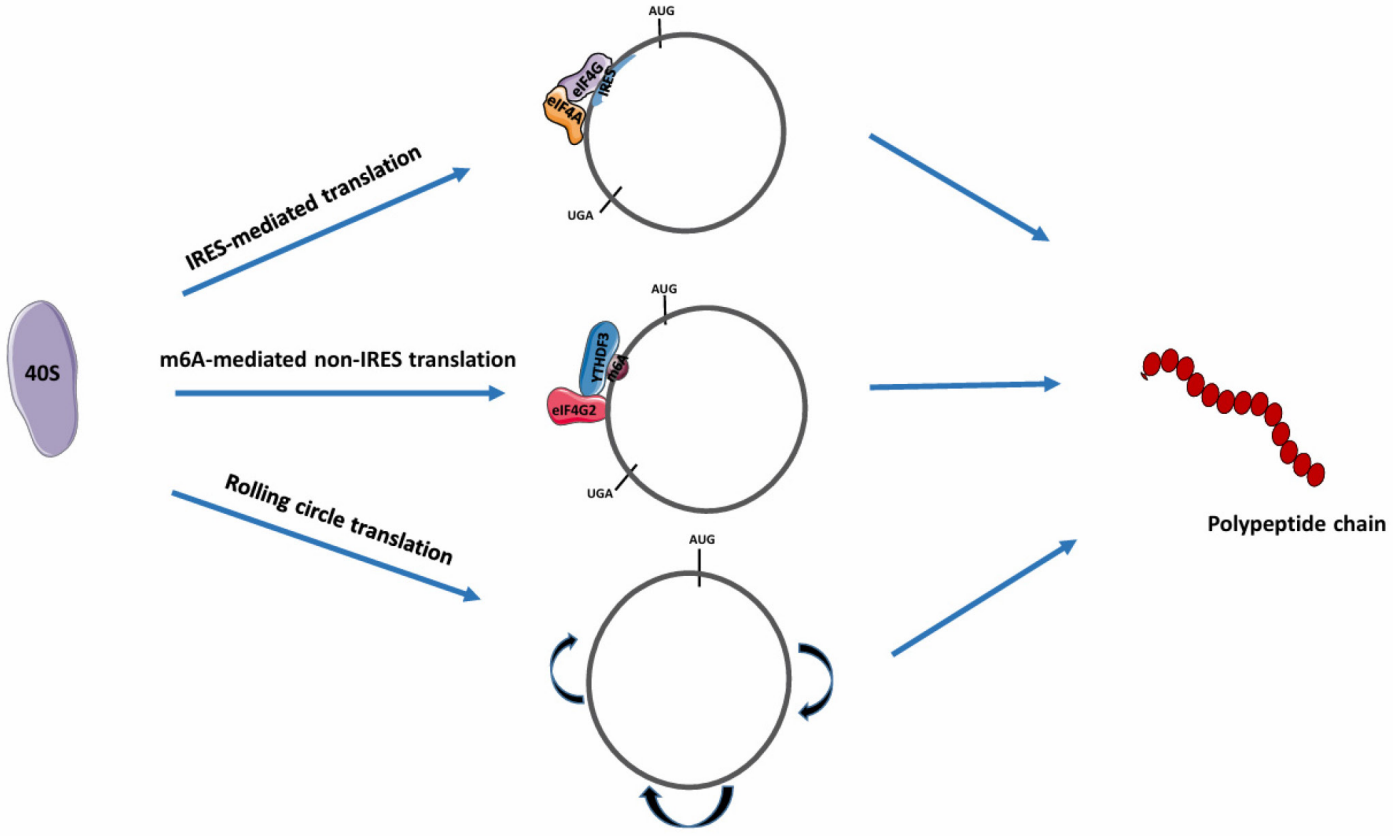
** Various mechanisms of circRNA translation.** CircRNA translation can proceed via different mechanisms depending on the internal features of the circRNA. In the presence of IRESs, IRES-like sequences such as m6A, or in their absence (for rolling circle translation), the respective eIFs recruit the 40S ribosome for circRNA translation. IRES, Internal ribosome entry site; eIF4G, Eukaryotic initiation factor 4G; eIF4A, Eukaryotic initiation factor 4A; eIF4G2, Eukaryotic initiation factor 4G2; m6A, N6-methyladenosine; YTHDF3, YTH N6-methyladenosine RNA Binding Protein 3. (Created in Servier Medical Art)

IRES are small, CU-rich, highly-structured nucleotide sequences of variable length that can recruit ribosomes (40S complex or ribosomal subunit) and translation initiation factors to facilitate cap-independent translation 34. Several studies have confirmed that initiation of translation in circRNAs occurs through an IRES-mediated mechanism 35, 36. In this form of translation, the binding of the ribosomal subunit to the IRES for translation is facilitated by the presence of eIF4G and eIF4A components on a specific domain of the IRES (Fig. 3) 33. Also, it has been reported that IRES-like short elements consisting of AU-rich hexamer sequences, located in the 3' UTR of linear mRNAs, can also drive circRNA translation 29. The IRES translation mechanism has been mainly observed in cells in severe stress or disease conditions 37. Fan et al demonstrated that some *trans*-acting factors can function as RBPs by binding to IRES elements to initiate circRNA translation 29. Chen et al maximized *in vitro* and *in vivo* translation of synthetic circRNAs by optimizing IRES elements, synthetic aptamers, 5' and 3' UTR, and vector topology, validating the role of IRES in circRNA translation initiation. Notably, increased *in vitro* circRNA translation was observed and may deliver more durable translation *in vivo* than mRNA protein expression 36. When combined with IRES, other RBPs can aid in the initiation of circRNA translation. Zhong et al reported that the DEAD-box helicase 3 (DDX3) interacted with the IRES sequence of circEZH2 to drive EZH2-92aa protein translation. However, the involvement of other regulatory elements remains unknown 38.

### Non-IRES translation mechanism

This mechanism involves N^6^-methyl adenosine (m^6^A) mediated protein translation and rolling circle translation.

In m^6^A-mediated protein translation, the m^6^A sites in the 5' UTR are positioned on short nucleotide sequences. The binding of YTHDF3, an m^6^A -binding protein to circRNAs, enables recruitment of the eIF4G2 and ribosome complex, leading to the initiation of circRNA translation (Fig. 3) 39. Tang et al reported that most circRNAs in male germ cells possess large ORFs with junction sequences containing significantly enriched m^6^A-modified start codons 40. These junction sequences enable peptide synthesis by encoding peptides, validating the mechanism of m^6^A-dependent protein translation. Proteins besides YTHDF3 can also facilitate m^6^A modification during circRNA translation. Through m^6^A modification, facilitated by IGF2BP1, the translation of circMAP3K4 produces circMAP3K4-455aa, a 455 amino acid (aa) protein, which is associated with the progression of hepatocellular carcinoma 41.

For rolling circle translation, circRNAs with an infinite ORF (i.e., without stop codons) that only contain coding sequences can be efficiently translated into a large protein concatemer via a rolling circle amplification mechanism 42. In this process, once translation is initiated, elongation proceeds indefinitely, and the ribosome does not have to continuously bind to the RNA (Fig. 3) 43. Abe et al showed that synthetic circRNAs with an infinite ORF prepared *in vitro* can be translated in living human cells devoid of particular elements such as IRES, a 3' poly-A tail, or a 5' cap structure 44. Notably, this process can produce a greater amount of protein compared to circRNAs with stop codons 29.

Although the protein-encoding potential of circRNAs containing IRESs has been deciphered, the fundamental principle of circRNA translation remains an open question. A thorough understanding of the mechanisms that drive circRNA translation is thus essential for developing efficient circRNA therapeutics capable of overcoming mRNA's fleeting nature and potentially exceeding the translation capabilities of mRNA 36.

## Bioinformatic tools and experimental techniques for detecting translatable circRNAs

The identification and study of circRNA-encoded proteins have been facilitated by recent advances in computational/bioinformatic tools (Table 1). Although bioinformatics analysis is useful, experimental techniques are necessary to provide evidence of the protein-encoding ability of most circRNAs (Table 2).

## Functions and characteristics of proteins expressed from circRNAs

Our current understanding of the characteristics and functions of circRNA-encoded proteins has been broadened due to extensive evidence from recently published studies confirming their roles in various disease pathogenesis, such as cancer metastasis, cardiac and neurological diseases. The unusual stability of circRNAs, which results from their circular structure, allows for a longer expression of the proteins they encode 45, 46. More so, circular RNAs are tissue-specific, which implies that the expression of the respective circRNA-encoded protein is exclusive to a particular tissue and can be targeted specifically 47, 48. This suggests a prominent role of circular RNAs in affecting cellular physiology within specific tissues, serving as potential biomarkers for tissue-related diseases. Most importantly, most circRNAs originate from the nucleus but are transported to the cytoplasm, where they are subsequently translated into proteins that modulate various cellular pathways and physiology. In this section, we focus on a few of these circRNA-encoded proteins and their respective roles in disease progression.

### Roles of circRNA-encoded proteins in cancer progression

**Figure 4:**
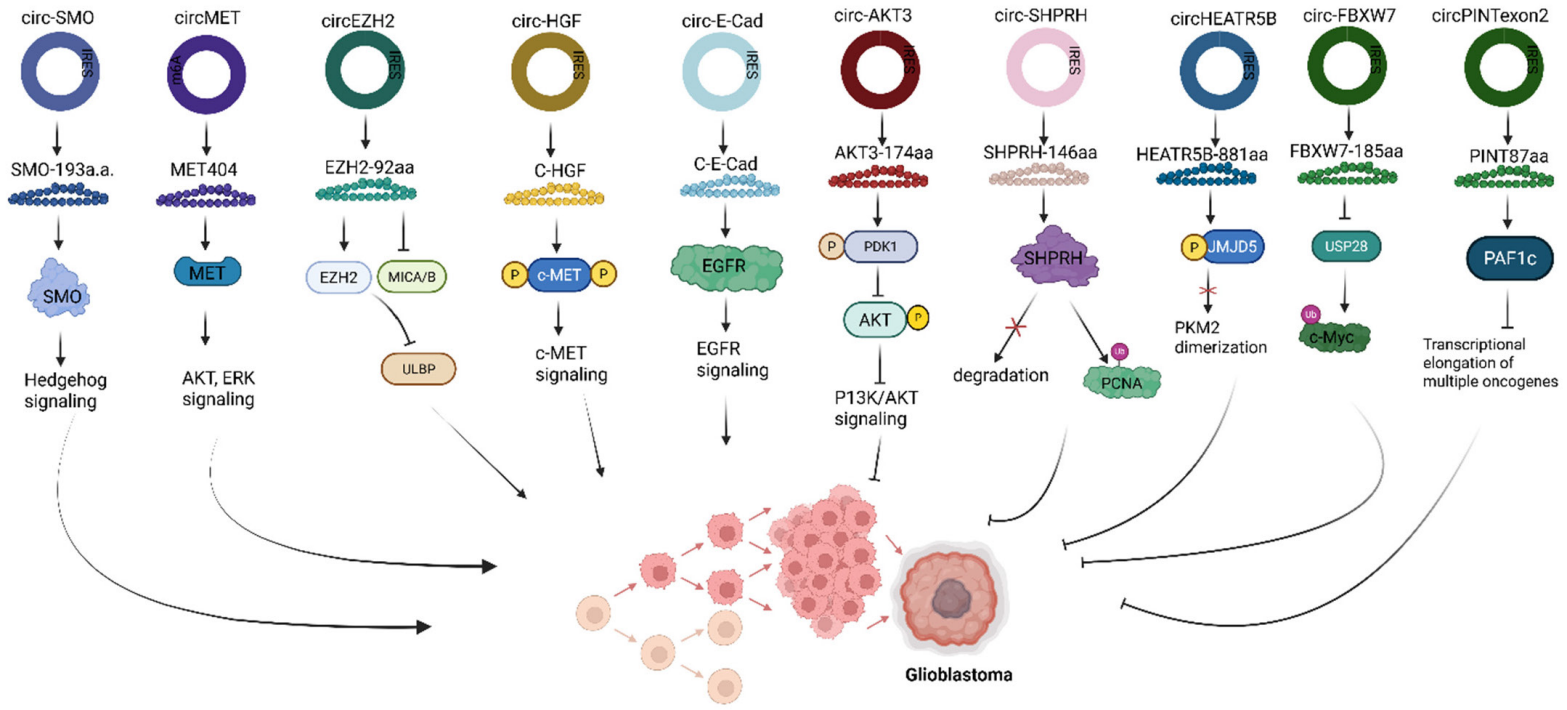
** Roles of translatable circRNAs in glioblastoma.** Circ, Circular RNA; IRES, Internal ribosome entry site; SMO, G protein‑coupled‑like receptor smoothened; MET, Mesenchymal to epithelial transition; EZH2, Enhancer of Zeste Homolog 2; MICA/B, Major histocompatibility complex class I polypeptide-related sequence A/B; ULBP, UL16-binding protein; c-HGF, Cell-surface hepatocyte growth factor; c-MET, Cell-surface MET receptor; E-Cad, E-cadherin; EGFR, Epidermal growth factor receptor; PDK1, 3‑phosphoinositide‑dependent kinase 1; AKT, Protein kinase B; P13K/AKT, Phosphoinositide 3-Kinase/ Protein Kinase B; SHPRH, SNF2 histone linker PHD RING helicase; PCNA, proliferating cell nuclear antigen; HEATR5B, HEAT repeat containing 5B; JMJD5, Jumonji‑C domain‑containing protein 5; PKM2, Pyruvate Kinase M2; FBXW7, F‑box and WD repeat domain containing 7; USP28, ubiquitin specific peptidase 28; c-Myc, Cellular myelocytomatosis oncogene; PINT, p53‑induced transcript; PAF1c, Polymerase‑associated factor complex. Reproduced with permission from REF 24, CC BY 4.0 (https://creativecommons.org/licenses/by/4.0) (Created in https://BioRender.com)

Circular RNA-encoded proteins can either function to inhibit or promote cancer tumor pathogenesis. For example, in glioblastoma, C-E-Cad, a 254 amino acid protein variant, encoded by the oncogenic circRNA, circ-E-Cad, is among the few circRNAs found to be upregulated in tumorigenic cells (Fig. 4) 49. C-E-Cad activates the EGFR signaling pathway by binding to the EGFR CR2 domain, leading to glioblastoma tumorigenesis 49. In another study, lending credence to the regulatory role of circRNA-encoded protein, highlights the significance of the Hedgehog pathway in driving tumorigenesis of many cancers, including glioblastoma. SMO-193aa, encoded by the circRNA circular SMO (circ-SMO), was shown to be crucial in the signaling activation of the Hedgehog pathway in glioblastoma 50. Circ-SMO is produced from exons 3 - 6 of the SMO gene, translating into a novel protein SMO-193aa via the IRES-mediated circRNA translation mechanism (Fig. 4). Mechanistically, SMO-193aa, by sharing most of its predicted five-domain transmembrane sequence with SMO, increases SMO cholesterol modification by binding to SMO, thus inhibiting the action of Ptch1 protein 50. Consequently, the Gli1 protein is activated for Shh downstream signaling. Fused in sarcoma (FUS), a downstream Gli1 protein target, is an RBP that has been shown to positively regulate the backsplicing of circ-SMO 51, promotes circ-SMO transcription. The network of Shh-Gli1-FUS-SMO-193aa forms a cycle that sustains HH signaling activation, resulting in glioblastoma progression 50.

**Figure 5:**
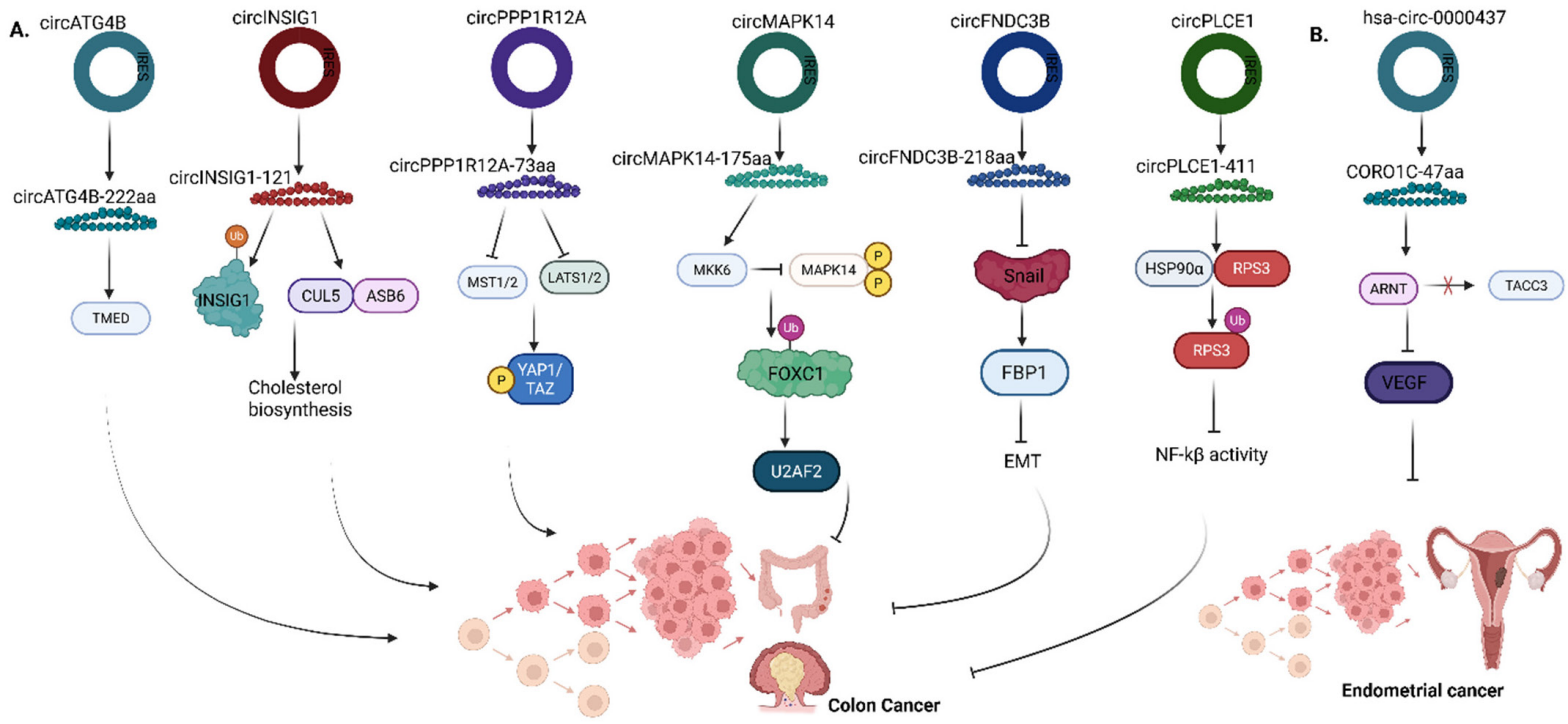
Roles of translatable circRNAs in the pathogenesis of **(A)** Colon cancer **(B)** Endometrial cancer. ATG4B, Autophagy Related 4B Cysteine Peptidase; TMED, Transmembrane Emp24 Domain-containing protein; INSIG1, Insulin Induced Gene 1; CUL5-ASB6 Complex, Cullin 5 - Ankyrin Repeat and SOCS Box containing protein 6; PPP1R12A - Protein Phosphatase 1 Regulatory Subunit 12A; MST1/2, Mammalian STE20-like protein kinase 1 and 2; LATS1/2, Large Tumor Suppressor Kinase 1 and 2; YAP1/TAZ, Yes-associated protein 1/Transcriptional coactivator with PDZ binding domain; MAPK14, Mitogen-activated protein kinase 14; MKK6, Mitogen-activated protein kinase kinase 6; FOXC1, Forkhead box C1; U2AF2, U2 small nuclear RNA auxiliary factor 2; FNDC3B, Fibronectin type III domain‑containing protein 3B; FBP1, Fructose-1,6-bisphosphatase; EMT, Epithelial‑mesenchymal transition; PLCE1, Phospholipase C Epsilon 1; HSP90α, heat‑shock protein 90α; RPS3, ribosomal protein S3; NF-Κb, Nuclear factor kappa-light-chain-enhancer of activated B cells; CORO1C - Coronin 1C; ARNT, Aryl hydrocarbon receptor nuclear translocator; TACC3, Transforming acidic coiled-coil containing protein 3; VEGF, Vascular endothelial growth factor. Reproduced with permission from REF 24, CC BY 4.0 (https://creativecommons.org/licenses/by/4.0) (Created in https://BioRender.com)

Autophagy is one of the key processes that induces chemoresistance, and enhances cancer cell survival. Previous studies have shown that the expression of a key autophagy-related factor known as ATG4B, significantly increased cancer chemoresistance in gastric, colorectal and colon cancers 52-54. ATG4B acts by cleaving the MAP1LC3/LC3 complex to form LC3-I and is subsequently bound to phosphatidylethanolamine (PE) to generate lipidated LC3-II on phagophore membranes 55-57. In colorectal cancer (CRC), the expression of circATG4B-222aa, encoded by circATG4B, was directly related to poor prognosis in patients with CRC who were treated with oxaliplatin. CircATG4B-222aa directly binds to TMED10, preventing TMED10 interaction with ATG4B, thus increasing autophagy, which triggers oxaliplatin resistance, thereby reducing the chemosensitivity of CRC cells (Fig. 5a) 56.

**Figure 6:**
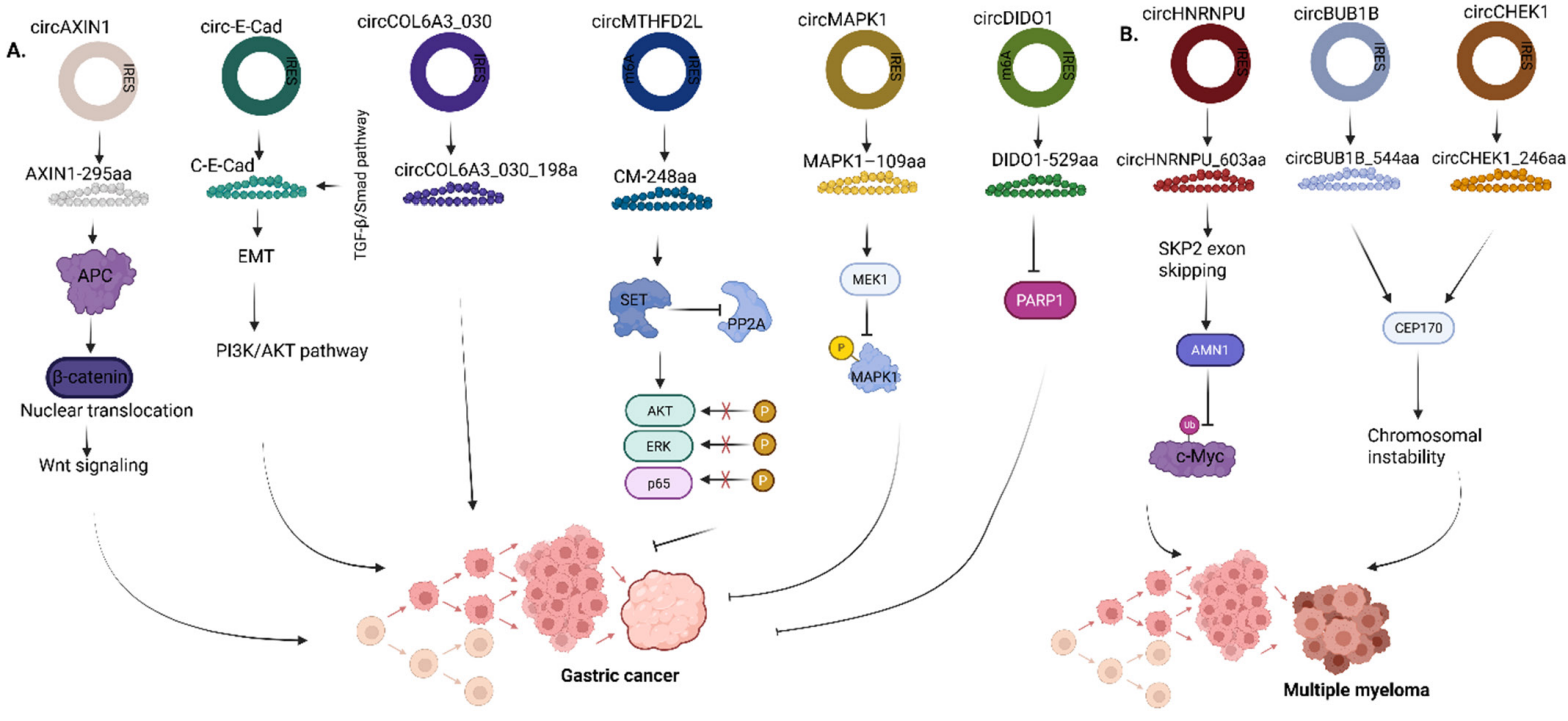
Roles of circRNA-encoded proteins in **(A)** Gastric cancer **(B)** Multiple myeloma. AXIN1, Axis inhibition protein 1; APC, Adenomatous polyposis coli; ERK, Extracellular signal-regulated kinase; MAPK1, Mitogen-activated protein kinase 1; MEK1, Mitogen-activated protein kinase kinase 1; DIDO1, Death inducer-obliterator 1; PARP1, Poly (ADP-Ribose) Polymerase 1; HNRNPU, Heterogeneous nuclear ribonucleoprotein U; SKP2, S-phase kinase-associated protein 2; AMN1, Aminopeptidase N 1; BUB1B, BUB1 mitotic checkpoint serine/threonine kinase B; CHEK1, Checkpoint kinase 1; CEP170, Centrosomal protein 170. Reproduced with permission from REF 24, CC BY 4.0 (https://creativecommons.org/licenses/by/4.0) (Created in https://BioRender.com)

In gastric cancer, AXIN1-295aa, encoded by circAXIN1, functions as an oncogenic protein by interacting with adenomatous polyposis coli (APC) competitively (Fig. 6a) 58. Functionally, APC forms a complex with glycogen synthase kinase 3 protein (GSK3), casein kinase 1 (CK1) and AXIN protein to recruit E3 ubiquitin ligase β-trcp to degrade β-catenin 59. However, the competitive interaction with AXIN1-295aa leads to the dysregulation of the Wnt signaling pathway, thus allowing β-catenin to effectively translocate into the cellular nucleus, binding to the transcription factors (TCF/LEF) and activating downstream signaling, which promotes the progression and metastasis of gastric cancer cells 58.

In multiple myeloma (MM), Tang et al discovered that a novel peptide, circHNRNPU_603aa, which enhances the progression of MM, is encoded by the circular RNA, circHNRNPU (Fig. 6b) 60. Analysis of the putative ORF of circHNRNPU on circRNA database showed that it contains an IRES sequence (from +201 to +374) and the ORF can potentially encode a 603-aa peptide, thus named, circHNRNPU_603aa. To determine if circHNRNPU_603aa promotes MM progression, colony formation assays, MTT and cell cycle assays were performed. The results of these assays indicated that circHNRNPU_603aa promoted MM cell proliferation and clonal expansion. Mechanistically, following the secretion of circHNRNPU, which encodes circHNRNPU_603aa in the bone marrows by MM cells, through the RNA-binding RGG-box region, circHNRNPU_603aa significantly regulates backsplicing of SKP2 exon and activates AMN1 to stabilize c-myc, thereby competitively inhibiting c-Myc ubiquitin (Fig. 6b) 60.

**Figure 7:**
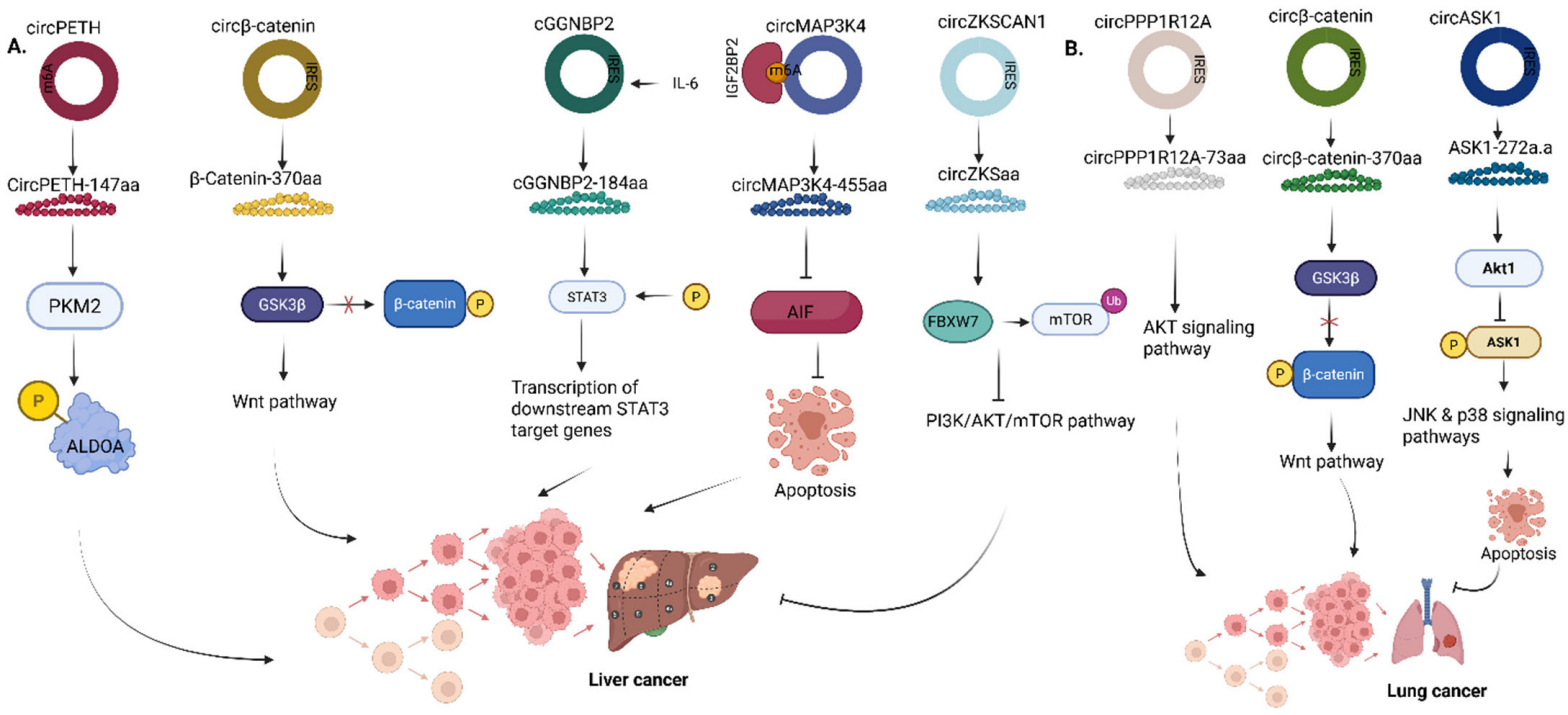
Roles of circRNA-encoded proteins in **(A)** Liver cancer **(B)** Lung cancer. PKM2, Pyruvate kinase M2; ALDOA, Aldolase A; GSK3β, Glycogen synthase kinase 3β; GGNBP2, Gamma-glutamyl-glycine beta-cytosine peptidase 2; STAT3, Signal transducer and activator of transcription 3; AIF, Apoptosis-inducing factor; MAP3K4, Mitogen-activated protein kinase kinase kinase 4; mTOR, Mechanistic target of rapamycin; ASK1, Apoptosis signal-regulating kinase 1. Reproduced with permission from REF.^24^, CC BY 4.0 (https://creativecommons.org/licenses/by/4.0) (Created in https://BioRender.com)

In liver cancer, the circRNA, circZKSCAN1, encodes a secretory protein located in the liver, circZKSaa, which is highly significant in the inhibition of proliferation of hepatocellular carcinoma (HCC) cells (Fig. 7a) 61. The phosphoinositide-3-kinase (P13K)/AKT signaling pathway is an important pathway involved significantly in the regulation of various cellular processes, including cell metabolism, angiogenesis, cell proliferation and viability 62. Irregularities in the P13K/AKT signaling pathway have been associated with the onset of different types of cancers, diabetes, inflammatory and autoimmune diseases, cardiovascular and neurological diseases 63-66. circZKSaa overexpression inhibits HCC tumor metastasis by interacting with mTOR and also facilitating the interaction of FBXW7 with mammalian target of rapamycin (mTOR), which is one of the downstream members of the Akt pathway (Fig. 7a) 61.

**Figure 8:**
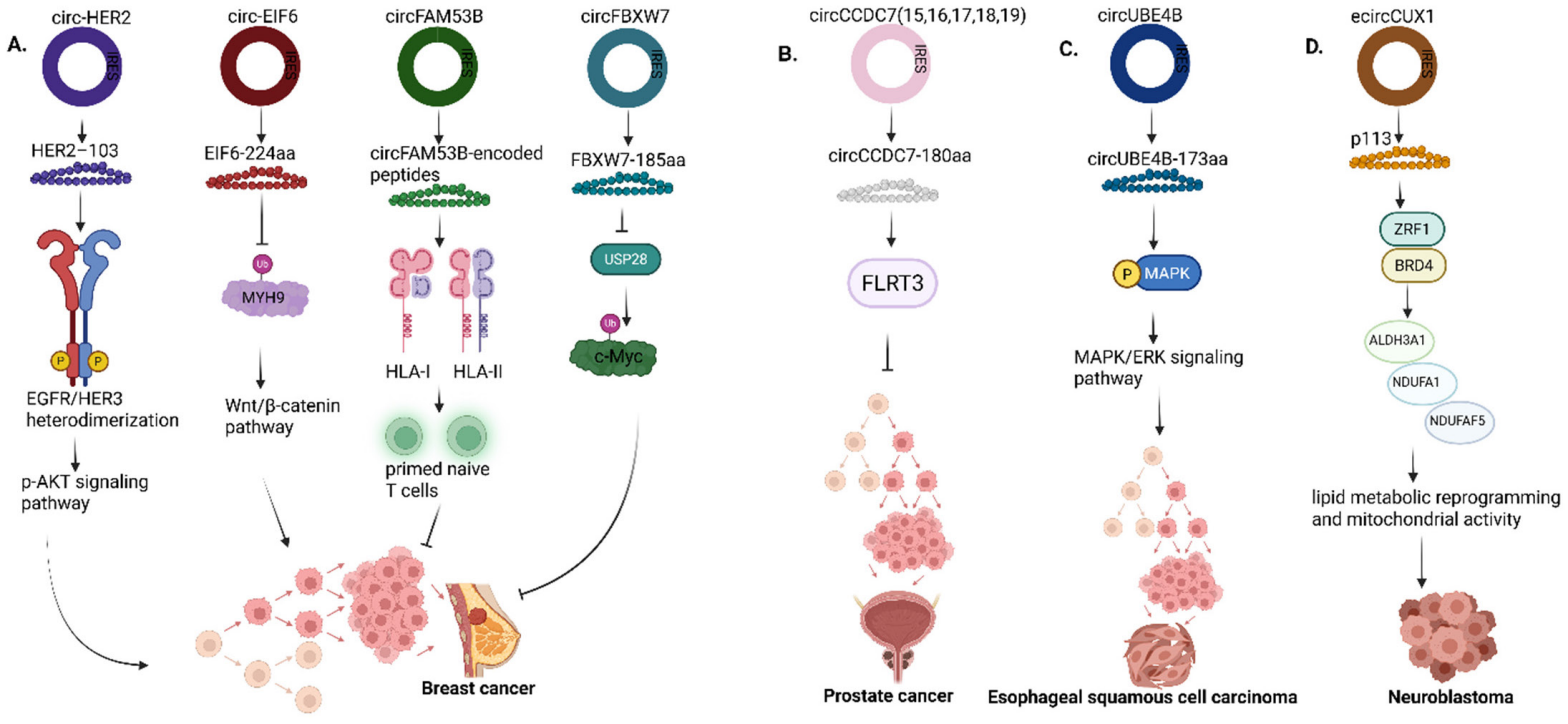
Roles of circRNA-encoded proteins in **(A)** Breast cancer **(B)** Prostate cancer **(C)** Esophageal squamous cell carcinoma **(D)** Neuroblastoma. HER2, Human epidermal growth factor receptor 2; EGFR, Epidermal growth factor receptor; HER3, Human epidermal growth factor receptor 3; EIF6, Eukaryotic translation initiation factor 6; MYH9, Myosin Heavy Chain 9; FAM53B, Family with sequence similarity 53 member B; HLA I & II, Human leukocyte antigen I & II; CCDC7, Coiled-coil domain containing 7; FLRT3, Fibronectin leucine rich transmembrane protein 3; UBE4B, Ubiquitination factor E4B; MAPK, Mitogen-activated protein kinase; ERK, Extracellular signal-regulated kinases; ZRF1, Zuotin-related factor 1; BRD4, Bromodomain containing protein 4; ALDH3A1, Aldehyde dehydrogenase 3 family member A1; NDUFA1, NADH:Ubiquinone oxidoreductase subunit A1, NDUFAF5, NADH:Ubiquinone oxidoreductase complex assembly factor 5. Reproduced with permission from REF 24, CC BY 4.0 (https://creativecommons.org/licenses/by/4.0) (Created in https://BioRender.com)

In breast cancer, EIF6-224aa encoded by circ-EIF6 was expressed endogenously in triple-negative breast cancer (TNBC) cells and tissues using a specific target antibody 67. Although the KD of circ-EIF6 alleviated tumor cell progression of TNBC, the study showed that EIF6-224aa activates the Wnt/beta-catenin pathway via interaction with MYH9 protein (Fig. 8a). In the canonical Wnt pathway, the binding of the Wnt proteins to its receptors causes the release of β-catenin and its translocation into the nucleus, leading to the activation of downstream target genes such as CCND1, c-Myc, etc. involved in cell proliferation and migration. Conversely, the absence of Wnt proteins causes the ubiquitination of β-catenin by E3 ubiquitin ligase, and thus β-catenin cannot interact with TCF/LEF, which represses the expression of downstream target genes 59, 68. Functionally, it was validated that EIF6-224aa overexpression decreased MYH9 ubiquitination, thereby shielding MYH9 from proteasomal degradation, and the expression of MYH9 correlates with the activation of the Wnt/beta-catenin pathway, as shown through the upregulation of downstream target genes such as CCND1, AXIN2, NKD1, etc, thereby leading to TNBC progression.

Finally, in prostate cancer, Wang et al showed that the circular RNA circCCDC7 (15,16,17,18,19) derived from exons 15 to 19 of the CCDC gene encodes a novel protein, circCCDC7-180aa, which functions as a tumor suppressor in prostate cancer by upregulating Fibronectin leucine-rich transmembrane protein 3 (FLRT3) (Fig. 8b) 69. FLRT3 has already been linked to the regulation of endothelial cell survival stimulated by VEGF signaling, cell migration and tube formation in endothelial cells and neuronal cell outgrowth and morphogenesis 69-71.

### Roles of circRNA-encoded proteins in neurological and cardiac diseases

**Figure 9:**
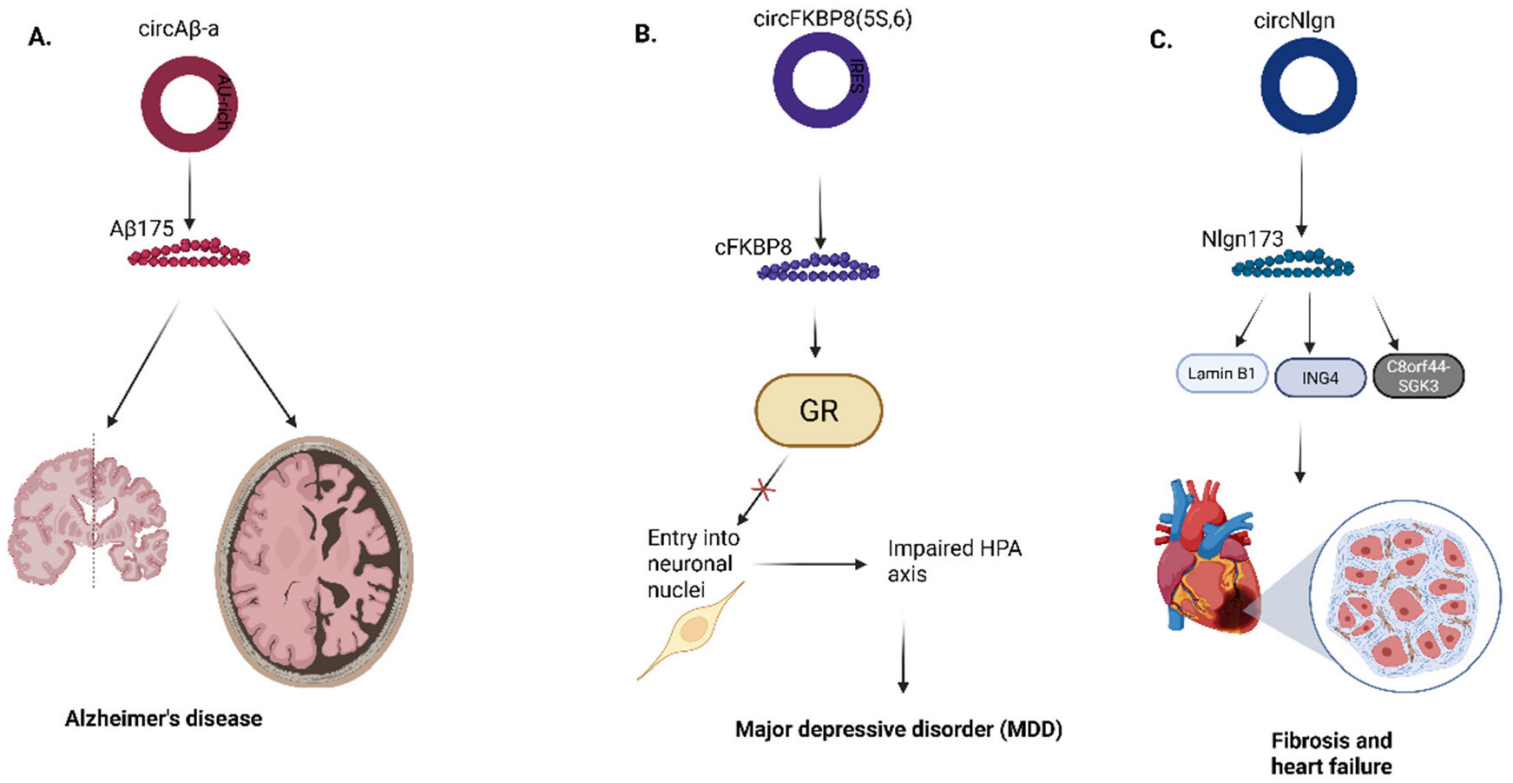
Roles of circRNA-encoded proteins in **(A)** Alzheimer's disease **(B)** Major depressive disorder **(C)** Cardiac disease. Aβ, amyloid beta; FKBP8, FK506-binding protein 8; GR, Glucocorticoid receptor; HPA, Hypothalamic - pituitary - adrenal axis; Nlgn, Neuroligin; ING4, Inhibitor of growth family member 4; C8orf44-SGK3, Chromosome 8 open reading frame 44 - Serum/Glucocorticoid regulated kinase 3 fusion. Reproduced with permission from REF 24, CC BY 4.0 (https://creativecommons.org/licenses/by/4.0) (Created in https://BioRender.com)

Compared to the research on circRNA-encoded proteins in the pathogenesis of various types of cancers, few works have been done to decipher their roles in the pathogenesis of neurological and cardiac diseases due to several limitations. Mo et al discovered that a circular RNA, circAβ-a, which harbors the Aβ-coding region of the APP gene, encodes a novel Aβ-containing Aβ175 polypeptide (Fig. 9a) 72. Aβ175 protein is processed into Aβ peptides, significant in Alzheimer's disease pathology. In this study, an alternative route for Aβ peptide production was confirmed because the Aβ175 contains β and γ-secretase cleavage sites; thus, there is a high probability of amyloid beta peptide production from Aβ175 through the cleavage of the β- and γ-secretase 72.

Recently, in the first study conducted on the role of circRNA-encoded proteins in the pathogenesis of major depressive disorder (MDD), Jiao et al showed that a circRNA termed circFKBP8(5S,6), which encodes a 127 aa protein termed cFKBP8, is significantly upregulated in stressed neuroblastoma cell lines and plasma neuronal-derived exosomes of human MDD patients. They further proved that cFKBP8 contributes to dysfunction of the hypothalamus-pituitary-adrenal (HPA) axis by preventing nuclear translocation of the glucocorticoid receptor (Fig. 9b) 73.

Lastly, in cardiac diseases, Du et al identified a circular RNA, circNlgn, produced by neuroglin that encodes a novel protein isoform, Nlgn 173, which promotes heart failure and cardiac remodeling (Fig. 9c) 74. Nlgn173 contains a binding site for LaminB1, a protein located within the nuclear membrane, which aids in binding transcription factors 75. The nuclear translocation of Nlgn173 is achieved via the interaction of LaminB1 with Nlgn173, where it functions as a transcription factor 74. Past studies elucidated that SGK3 potentially interacts with and phosphorylates GSK-3β at serine-9, to form the pS9-GSK3β complex, which inactivates GSK3 activity and induces hypertrophic cardiomyopathy 76, 77. On the other hand, ING4 can also induce cellular apoptosis and inhibit tumor cell growth through its interaction with p53 or BCL2 family proteins 78, 79.

## Systematic circRNA-encoded protein discovery

The process of circRNA-encoded protein discovery in tumor diseases is similar to that for non-tumor diseases, such as cardiac diseases. This process involves the integration of both experimental and bioinformatics techniques for efficient circRNA identification. CircRNA-encoded proteins can regulate the pathogenesis of tumor diseases by either inhibiting or promoting tumor metastasis (Table 3, Figs. 4-8).

First, RNAs are extracted from tissue samples obtained from tumors and control patients. To efficiently identify and quantify circRNAs, high-throughput RNA sequencing is then carried out, and, various bioinformatics analysis tools, such as find_circ or circExplorer, are employed to identify unique circRNAs based on their characteristic back-splice junctions (BSJs). Subsequently, the already identified circRNAs are validated using techniques such as RT-PCR or Sanger sequencing. Because circRNAs exhibit differential levels of expression in tumors and normal tissues, comparing both tissues allows for the identification and confirmation of differentially expressed circular RNAs (DeCRs). Finally, the predominant cellular localization of circRNA is ascertained using fluorescent *in situ* hybridization (FISH) or subcellular fractionation assay to give insights into its protein-encoding ability.

When studying circRNA-encoded proteins, it is necessary to predict the protein-encoding potential. The presence of an ORF sequence within the circRNA sequence indicates that a circRNA is indeed translatable 29. Thus, circRNA sequences can be analyzed using online databases and tools containing a repository of information on circRNA supported by experimental evidence. In the case of IRES-mediated translation, the ORFs contain internal IRES sequences that initiate protein translation. Ribosome profiling can be used to confirm the association of circRNAs with ribosomes, suggesting translation into proteins, and further validated by LC-MS, Co-IP, or western blotting (by directing specific antibodies to precise peptide terminal sequences).

The effect of knockdown of circRNA or its encoded protein using siRNAs or CRISPR/Cas technologies in animal models or selected cell lines is assessed based on changes in cell migration, proliferation, and invasion 80. Proper elucidation of the mechanism that drives the encoded protein to exert its tumor proliferative or inhibitory effect must be established. For example, Jiang et al found that MAPK1-109aa is significantly downregulated in patients with gastric cancer 81. Thus, overexpression of circMAPK1, acting through its encoded protein, MAPK1-109aa, inhibits metastasis of gastric tumor cells. MAPK1-109aa acts as a tumor inhibitor by binding to MEK1 (mitogen-activated protein kinase kinase), thereby inhibiting phosphorylation of MAPK1 and its downstream effectors 81.

## Circular RNA-encoded proteins as potential therapeutic targets

The discovery that circular RNA can actually encode various proteins that are implicated in mechanisms, pathways and signaling processes associated with various diseases, opened up a new window of opportunity towards their potential target for therapeutic intervention. This section explores some potential therapeutic targets associated with circRNA-encoded proteins and how they impact disease pathogenesis.

Glioblastoma is the most common form of brain cancer, and given that activated MET signaling/ MET alteration contributes significantly to glioblastoma 82, a thorough understanding of the MET signaling pathway is required to identify functional therapeutic targets to aid the development of effective treatment for this disease. Here, MET404, a 404-amino-acid encoded by the circular MET RNA, circMET, facilitates glioblastoma tumorigenesis, thus indicating its clinical relevance as a potential molecular target 30. In mice, abrogation of MET signaling was achieved through the genetic alteration of circMET, which reduced MET404 expression. MET 404 promotes tumor cell proliferation by directly binding to the MET β subunit, leading to the outright activation of the MET receptor and downstream effectors (Fig. 4a). Previously, HGF was the only known MET ligand that binds to the SEMA domain on MET 30, 83. This study revealed that MET 404 is also a MET ligand and binds to the (PSA+IPTI) domain on the MET receptor. Knockdown of the circMET gene was also shown to repress the expression of MET 404 in mice, while high expression of MET 404 further exacerbates glioblastoma tumorigenicity *in vivo* and *in vitro*. Thus, targeting MET 404 can lead to a potential treatment option for glioblastoma patients with MET hyperactivation. To confirm this, genetic ablation of circMET or the combined administration of MET404 antibody and onartuzumab, an FDA-approved traditional MET signaling inhibitor, significantly repressed MET signaling and improved cellular viability 30.

In a recent study, circPETH-147aa, encoded by circPETH (derived from exons 13-15 of the low-density lipoprotein receptor gene), was shown to enhance glycolysis and metastasis in HCC cells (Fig. 7a) 84. A druggable MEG pocket of circPETH-147aa was identified and was confirmed to interact with PKM2, ALDOA and HuR. Overexpression of circPETH-147aa increased ALDOA phosphorylation by PKM2, increasing HuR-dependent SLC43A2 mRNA stability, which enhances cytotoxic CD8+ T cells exhaustion and abrogates anti-HCC immunity. High-throughput virtual screening identified a small-molecule compound, Norathyriol, which is a natural product that effectively showed a profound interaction with the MEG pocket of circPETH-147aa and was validated experimentally. Taken together, administration of Norathyriol alone or in combination with anti-PD1 antibodies effectively strengthened the activity of CD8+ T cells in a mouse model of lung metastasis and liver orthotopic intrahepatic metastasis 84.

Furthermore, Wang et al discovered that a novel protein encoded by circASK1, ASK1-272a.a, is involved in lung adenocarcinoma (LUDA) by showing that circASK1 originated from a MAPK signaling gene 85. Given the reduced level of ASK1 in cells with acquired gefitinib resistance, it was chosen for further investigation. In *in vitro* experiments, KD of circASK1 made LUAD cells resistant to gefitinib treatment while circASK1 overexpression alleviated the gefitinib resistance of LUAD cells. Mechanistically, ASK1-272a.a activates ASK1 through competitive interaction with Akt1, thereby enhancing ASK1-mediated apoptosis through cellular susceptibility to gefitinib (Fig. 7b). Finally, ASK1-272a.a enhances the LUAD cells' sensitivity to gefitinib; therefore, ASK1-272a.a can act as a potential biomarker for tracking patients' response to gefitinib treatment.

Vaccination utilizing the protein-encoding ability of circRNAs has also shown remarkable improvement towards targeting cancer tumors by harnessing and activating cytotoxic T cells. This was shown in a study in which the *in vivo* administration of peptide and circRNA vaccines consisting of circFam53B-219 peptide and circFAM53B, respectively, in mice bearing breast cancer tumors or melanoma, activated tumor-antigen specific cytotoxic T cells, resulting in increased tumor lysis and effective tumor management (Fig. 8a) 45.

Finally, MDD is a devastating mental health disease whose pathogenesis remains vague and is not completely understood. One of the theories that has been linked to the onset of MDD is the impairment of the hypothalamic-pituitary-adrenal HPA axis mediated by the glucocorticoid receptor (GR). Jiao et al. explained how this dysfunction occurs through investigation of the translational ability of circFKBP8(5S,6), an upregulated circular RNA in the peripheral plasma of MDD patients. They clearly showed through a series of *in vitro*, and *in vivo* experiments in mice and macaques, that circFKBP8(5S,6)-encoded protein, cFKBP8, inhibited GR entry into neuronal nuclei, associated with the dysregulation of HPA axis and depression (Fig. 9b). Thus, can serve as a reliable therapeutic target and diagnostic biomarker for the treatment of MDD 73.

Therefore, circRNA-encoded proteins implicated in disease pathogenesis can be potentially targeted through genetic-based alteration, use of antibodies and small-molecule inhibitors; they have also been proven to be effective biomarkers, beneficial for monitoring various disease conditions. Taken together, the above examples highlight the clinical relevance of circular RNA-encoded proteins as potential diagnostic biomarkers and therapeutic targets for disease treatment.

## Limitations and controversies in circRNA-encoded protein research

Over the past one and a half decades, the field of circRNA biology has witnessed tremendous advancement regarding the detection and elucidation of their different physiological functions. Indeed, a significant amount of research has been carried out in this area. Despite this, we have to be aware of certain limitations and unsolved puzzles that still limit our understanding of this particular area of human biology.

Within the context of circRNA-encoded proteins implicated in CNS diseases, the sophisticated and heterogeneous nature of the CNS contributes to the varied expression of circRNAs in different brain regions, which can directly affect the accuracy of circRNA expression results. Also, clinical translation involves experimenting on live human brain tissues for proper validation of *in vitro* and *in vivo* experimental results to confirm these circRNAs as biomarkers. As it is unethical and nearly impossible to obtain live human brain tissues, especially from MDD patients, alternative techniques, such as the cerebral organoid technique, have been adopted 73, 86. However, these approaches do not reflect real-life depressive-like patterns seen in humans. This presents a major challenge to research that needs to be addressed urgently.

A much more significant bottleneck associated with circRNA-encoded protein research is the experimental validation of circRNA-encoded peptides. Although circRNAs are more stable than linear mRNAs, they are still poorly expressed compared to their linear counterparts, thus contributing to false positives that inadvertently affect accurate detection and quantification of circRNAs. For instance, the use of RiboSeq for the detection of circRNA translation is challenging, and without the use of appropriate controls, results can be misleading 15. Actually, a criterion for active translation depicted by a high-quality ribosome dataset is the presence of a strong triplet periodicity, otherwise known as phasing, along the regions/sequences where the ribosomes are bound 15. Therefore, to ensure reliable circRNA translation results, circRNA Riboseq results showing reads mapped to the backsplicing site must, as a matter of fact, show strong phasing and must be compared stringently to control RNAs which do not undergo translation, such as miRNA, snoRNA 15. Secondly, mass spectrometry is a widely adopted experimental technique for peptide identification; however, when it comes to unorthodox protein identification, such as those translated from circRNAs, rigorous analysis and checks are necessary to drastically minimize the risk of false discoveries. Therefore, the use of appropriate false discovery rate (FDR) control should be employed in these studies to efficiently validate circRNA translation 15.

Currently, there is still a huge debate amongst scientific researchers over the functional relevance of circRNA-encoded proteins. Despite the vast amount of published studies on circRNA-encoded proteins and their respective roles, a large skepticism still exists. The cap-independent mechanism of translation adopted by circRNAs, because of the absence of 5'cap, is highly inefficient due to sparse endogenous IRESs in the eukaryotic transcriptome 29, leading to low abundance of circRNA-encoded proteins; therefore, future research should aim to address this. There is a need to properly identify and validate all circRNAs that are indeed translatable using more sophisticated experimental and analytical platforms.

Another notable challenge with research on circRNA-encoded proteins is the detection of low-abundance circRNAs, including those that encode proteins. For example, there is evidence that trans-splicing byproducts of overexpression plasmids might have been misinterpreted as being translated from circRNAs 87. The researchers discovered that deletion of the 3' splicing site required for circZNF609 formation or *Alu* elements necessary for backsplicing generated neither circZNF609 nor its corresponding protein in the former case. However, surprisingly, in the latter case, while circZNF609 was not produced, the translated protein was detected. Further concurrent deletion of both splicing site and *Alu* elements generated neither circZNF609 nor its protein product. Thus, establishing that the protein product might have been a result of by-products of linear splicing and not necessarily from circular RNA backsplicing 87. Therefore, results from circRNA overexpression constructs should be interpreted with caution due to complications that can arise from overexpression systems. Most importantly, it should be noted that circRNAs possess similar sequences to their linear mRNA analogue, therefore conventional detection methods such as RT-qPCR may be faced with the challenge of non-specificity or insensitivity since genomic rearrangement does occur and the possibility for low-quality measurement exists.

In addition, interpretation of results from bulk circRNA RNA-seq of malignant tumor tissues compared to normal tissues, to determine differential circRNA expression, can also be misleading 88, 89. Employing a broader and more in-depth technique, such as spatial analyses and single-cell analyses, is essential to correctly interpret transcriptomic results. For instance, studies employing spatial analyses have shown that the circRNA, ciRS-7 (CDR1as), which was previously thought to be abundant in tumors compared to normal tissues while correlating with poor prognosis in diseased patients, was actually not expressed in specific colon cancer cells but in stromal cells within the tumor microenvironment. Thus, the high expression from the bulk tissue analysis resulted from the stromal cell expression and not from the colon cancer cells themselves 89. Generally, the expression of circRNAs, such as cirRS-7, is limited in proliferating cells, such as cancer cells but is more profound in quiescent cells, like muscle and stromal cells 88. This may lead to misinterpretations when studying patient samples using bulk RNA analysis only without considering varying expressions within the different cell types 88.

Finally, the clinical translation of circRNA-encoded proteins is still in its early stages. Development of antibodies or small-molecule inhibitors for circRNA-encoded proteins has to resolve the issue of immune rejection, and a thorough understanding of the safety of the inhibitor drug for human administration is crucial. Nevertheless, off-target effects present a major bottleneck for gene-therapy treatment to target endogenous circRNAs.

## Conclusion and future perspectives

Recent studies have highlighted the roles of circRNA-encoded proteins as potential clinical biomarkers for the diagnosis and treatment of several tumor and non-tumor diseases 19, 22. Nevertheless, there are important gaps in knowledge that must be addressed in the future to improve the clinical relevance of circRNA-encoded proteins. At present, knowledge of the mechanism of circRNA translation within cells is still vague. How several molecular components interact within the cell to drive circRNA translation must be studied. Of course, this will require a great deal of time and effort. The special characteristics of circRNA that direct mediatory translational mechanisms also remain an open question.

Furthermore, the research involving circRNA-encoded proteins in central nervous system (CNS) diseases is not clinically valuable because of the lack of appropriate model organisms that reflect real-life phenotypic characteristics of the diseases. This presents a serious impediment to research in this field. Although research using organoids represents a crucial step forward, certain limitations exist when using such technologies, as addressed in the previous section.

Given the impressive potential of artificial intelligence (AI), the incorporation of advanced AI tools in circRNA research presents an important opportunity to deeply understand the functional characteristics of circRNAs and address open questions. Therefore, future research should aim to integrate AI, bioinformatics and omics technologies in circRNA studies 5.

In summary, circRNA-encoded proteins are potentially important clinical biomarkers and therapeutic targets for the treatment of tumors and non-tumor diseases. To actualize the potential of circRNA-encoded proteins, more research involving large cohorts of patients is needed to validate their expression levels in different diseases. Extensive elucidation of the functional mechanisms of all circRNA-encoded proteins should be prioritized to enable actualization of their therapeutic potential, either as specific biomarkers or as targets for small molecules. This review calls for a global and collective effort towards the development and refinement of robust strategies for the identification of all possible circRNA-encoded proteins to fully understand their relevance, as well as refining targeted therapeutic techniques while establishing a universal standard in adopting circular RNA-encoded proteins as unique biomarkers for disease diagnoses.

## Figures and Tables

**Table 1:** Bioinformatic tools/databases and corresponding websites

CircRNA detection		
CIRCfinder	https://github.com/ YangLab/CIRCfinder	12
find_circ	https://github.com/marvin-jens/find_circ	90
MapSplice	http://www.netlab.uky.edu/p/bioinfo/MapSplice	91
CIRIquant	https://sourceforge.net/projects/ciri	92
CircExplorer2	https://github.com/YangLab/ CIRCexplorer2	16
CIRI2	https://sourceforge.net/ projects/ciri/files/CIRI2	93
UROBORUS	https://github.com/WGLab/UROBORUS	94
CIRI-Deep	https://github.com/gyjames/CIRI-deep	95
**Databases and Resources**		
CircBase	http://www.circbase.org/	50, 96
circInteractome	https:// circinteractome. nia.nih.gov/	97
CircNet	http://circnet.mbc.nctu.edu.tw/	98
TransCirc	https://www.biosino.org/transcirc/	99
CircRNADb	http://reprod.njmu.edu.cn/circrnadb	100
Circbank	www.circbank.cn	101, 102
ORF Finder	www.ncbi.nlm.nih.gov/gorf/gorf.html	24, 103
IRESfinder	https://github.com/xiaofengsong/IRESfinder	104
IRESbase	http://reprod.njmu.edu.cn/cgi‑bin/iresbase/index.php	105
IRESite	http://www.iresite.org	106
CPAT	http://lilab.research.bcm.edu/cpat/index.php	107, 108
**Protein-protein interactions**		
PEP-FOLD	https://bioserv.rpbs.univ-parisdiderot.fr/services/PEP-FOLD4/	109, 110
Phyre2	http://www.sbg.bio.ic/ac.uk/phyre2	111
Alphafold2	https://alphafold.com	101
i-TASSER	http://zhanglab.ccmb.med.umich.edu/I-TASSER	112
catRAPID	http://s.tartaglialab.com/page/catrapid_group	112
PEP-SiteFinder	http://bioserv.rpbs.univ-paris-diderot.fr/PEP-SiteFinder	110
ZDOCK	http://zdock.umassmed.edu	113
PPA-Pred2	http://www.iitm.ac.in/bioinfo/PPA_Pred/	114, 115
ClusPro2	https://cluspro.org	101
Peptiderive	http://rosie.rosettacommons.org/peptiderive	116, 117

**Table 2:** Experimental techniques for detecting translatable circRNAs

Technique	Description	References
RNA seq, m^6^A-seq, RNC-seq, YTHDF2 RIP-seq, ribosome profiling, ribosome immunoprecipitation, Ribosome fragment analysis, nanopore sequencing, ribosome affinity purification	To identify circular RNAs with encoding functions.	30, 118
Northern blotting	To validate circRNA expression and characterize the output of the overexpression of circRNAs using plasmids/vectors	20, 119
Sanger sequencing	To confirm the BSJ site of circRNAs	120
High-performance liquid chromatography-mass spectrometry (LC-MS), co-immunoprecipitation (co-IP), reciprocal immunoprecipitation, immunofluorescence staining	To provide direct evidence by identifying specific peptides	38, 58, 121
ELISA, western blotting	To detect putative peptides or proteins	30, 122
Dual luciferase reporter assay	For experimental evaluation of IRES/m6A activity of translatable circRNAs.	114
CCK-8, colony formation, EdU, Transwell and wound healing assays	For *in vitro* assessment of the influence of peptides on cell growth, migration, and invasion	81
Tagged RNA affinity purification (TRAP) assay and RNA immunoprecipitation (RIP) assay.	To identify/verify interaction between individual proteins and RNA.	112
si/shRNAs and CRISPR-Cas13 system.	To deplete circRNAs by targeting BSJs	20, 122
Plasmid and viral expression	For circRNA overexpression	18, 45, 73

**Table 3:** Translatable circRNAs and their roles in disease pathogenesis

CircRNA	Protein	Disease	Translation mechanism	Protein interaction	References
circFAM53B	**circFAM53B-encoded peptides** (Upregulated)	Breast cancer	IRES-mediated	HLA-I and HLA-II molecules	45
circ-EIF6	**EIF6-224aa** (Upregulated)	Triple Negative Breast cancer (TNBC)	IRES-mediated	MYH9	67
circFBXW7	**FBXW7-185aa** (Downregulated)	TNBC	IRES-mediated	USP28	123
circ-HER2	**HER2-103** (Upregulated)	TNBC	IRES-mediated	EGFR/HER3	124
circNlgn	**Nlgn173** (Upregulated)	Cardiac disease	-	LaminB1, ING4 & C8orf44-SGK3	74
circPLCE1	**circPLCE1‑411** (Downregulated)	Colorectal cancer	IRES-mediated	HSP90α/RPS3 complex	107
circPPP1R12A	**circPPP1R12A-73aa** (Upregulated)	Colorectal cancer	-	YAP1	125
circFNDC3B	**circFNDC3B-218aa** (Downregulated)	Colorectal cancer	IRES-mediated	Snail	121
circINSIG1	**circINSIG1-121** (Upregulated)	Colorectal cancer	IRES-mediated	INSIG1	126
circATG4B	**circATG4B-222aa** (Upregulated)	Colorectal cancer	IRES-mediated	TMED10	56
circMAPK14	**circMAPK14‑175aa** (Downregulated)	Colorectal cancer	IRES-mediated	MKK6	127
hsa-circ-0000437	**CORO1C-47aa** (Downregulated)	Endometrial cancer	IRES-mediated	ARNT	122
circAXIN1	**AXIN1-295aa** (Upregulated)	Gastric cancer	IRES-mediated	APC	58
circMAPK1	**MAPK1-109aa** (Downregulated)	Gastric cancer	IRES-mediated	MEK1	81
circ-E-Cad	**C-E-Cad** (Upregulated)	Gastric cancer	IRES-mediated	p-AKT and p-PDK1	128
circDIDO1	**DIDO1-529aa** (Downregulated)	Gastric cancer	IRES-mediated/ m6A modification	PARP1	112
circMTHFD2L	**CM-248aa** (Downregulated)	Gastric cancer	IRES-mediated and m6A modification	SET	101
circCOL6A3_030	**circCOL6A3_030_198aa** (Upregulated)	Gastric cancer	IRES-mediated	-	129
circ-SMO	**SMO-193a.a.** (Upregulated)	Glioblastoma	IRES-mediated	SMO	50
circ-SHPRH	**SHPRH-146aa** (Downregulated)	Glioblastoma	IRES-mediated	SHPRH	130
circ-AKT3	**AKT3-174aa** (Downregulated)	Glioblastoma	IRES-mediated	p-PDK1	131
circEZH2	**EZH2-92aa** (Upregulated)	Glioblastoma	IRES-mediated	MICA/B	38
circMET	**MET404** (Upregulated)	Glioblastoma	m6A modification	MET	30
circ-E-Cad	**C-E-Cad** (Upregulated)	Glioblastoma	IRES-mediated	EGFR	49
circ-FBXW7	**FBXW7-185aa** (Downregulated)	Glioblastoma	IRES-mediated	USP28	132
circ-HGF	**C-HGF** (Upregulated)	Glioblastoma	IRES-mediated	c-MET	133
CircPINTexon2	**PINT87aa** (Downregulated)	Glioblastoma	IRES-mediated	PAF1c	134
circHEATR5B	**HEATR5B‑881aa** (Downregulated)	Glioblastoma	IRES-mediated	JMJD5	114
circPETH	**CircPETH-147aa** (Upregulated)	Hepatocellular carcinoma (HCC)	m6A modification	PKM2, ALDOA, HuR	84
circMAP3K4	**circMAP3K4‑455aa** (Upregulated)	HCC	m6A modification	AIF	41
circZKSCAN1	**CircZKSaa** (Downregulated)	HCC	IRES-mediated	mTOR	61
circβ-catenin	**β-Catenin-370aa** (Upregulated)	Liver cancer	IRES-mediated	GSK3β	135
cGGNBP2	**cGGNBP2-184aa** (Upregulated)	Intrahepatic cholangiocarcinoma (ICC)	IRES-mediated	STAT3	136
circASK1	**ASK1-272a.a** (Downregulated)	Lung cancer	IRES-mediated	Akt1	85
circPPP1R12A	**circPPP1R12A-73aa** (Upregulated)	Lung cancer	-	p-AKT	137
circβ‑catenin	**circβ‑catenin‑370aa** (Upregulated)	Non-small cell Lung cancer	IRES-mediated	GSK3β	138
circHNRNPU	**circHNRNPU_603aa** (Upregulated)	Multiple myeloma	IRES-mediated	AMN1	60
circBUB1B	**circBUB1B_544aa** (Upregulated)	Multiple myeloma	IRES-mediated	CEP170	139
circCHEK1	**circCHEK1_246aa** (Upregulated)	Multiple Myeloma	IRES-mediated	CEP170	140
circCCDC7(15,16,17,18,19)	**circCCDC7-180aa** (Downregulated)	Prostate cancer	IRES-mediated	FLRT3	69
circAβ-a	**Aβ175** (Upregulated)	Alzheimer's disease	IRES-like elements (AU-rich)	-	72
circUBE4B	**circUBE4B‑173aa** (Upregulated)	Esophageal squamous cell carcinoma	IRES-mediated	MAPK1	141
ecircCUX1	**p113** (Upregulated)	Neuroblastoma	IRES-mediated	ZRF1 and BRD4	117
